# Heart–brain interactions shape somatosensory perception and evoked potentials

**DOI:** 10.1073/pnas.1915629117

**Published:** 2020-04-27

**Authors:** Esra Al, Fivos Iliopoulos, Norman Forschack, Till Nierhaus, Martin Grund, Paweł Motyka, Michael Gaebler, Vadim V. Nikulin, Arno Villringer

**Affiliations:** ^a^Department of Neurology, Max Planck Institute for Human Cognitive and Brain Sciences, 04103 Leipzig, Germany;; ^b^MindBrainBody Institute, Berlin School of Mind and Brain, Humboldt-Universität zu Berlin, 10099 Berlin, Germany;; ^c^Center for Stroke Research Berlin (CSB), Charité – Universitätsmedizin Berlin, 10117 Berlin, Germany;; ^d^International Max Planck Research School on the Life Course, Max Planck Institute for Human Development, 14195 Berlin, Germany;; ^e^Experimental Psychology and Methods, Faculty of Life Sciences, University of Leipzig, 04109 Leipzig, Germany;; ^f^Neurocomputation and Neuroimaging Unit, Department of Education and Psychology, Freie Universität Berlin, 14195 Berlin, Germany;; ^g^Faculty of Psychology, University of Warsaw, 00-927 Warsaw, Poland;; ^h^Institute of Cognitive Neuroscience, National Research University Higher School of Economics, 101000 Moscow, Russia

**Keywords:** consciousness, somatosensory awareness, body–brain interaction, EEG, rhythms

## Abstract

Even though humans are mostly not aware of their heartbeats, several heartbeat-related effects have been reported to influence conscious perception. It is not clear whether these effects are distinct or related phenomena, or whether they are early sensory effects or late decisional processes. Combining electroencephalography and electrocardiography, along with signal detection theory analyses, we identify two distinct heartbeat-related influences on conscious perception differentially related to early vs. late somatosensory processing. First, an effect on early sensory processing was found for the heartbeat-evoked potential (HEP), a marker of cardiac interoception. The amplitude of the prestimulus HEP negatively correlated with localization and detection of somatosensory stimuli, reflecting a more conservative detection bias (criterion). Importantly, higher HEP amplitudes were followed by decreases in early (P50) as well as late (N140, P300) somatosensory-evoked potential (SEP) amplitudes. Second, stimulus timing along the cardiac cycle also affected perception. During systole, stimuli were detected and correctly localized less frequently, relating to a shift in perceptual sensitivity. This perceptual attenuation was accompanied by the suppression of only late SEP components (P300) and was stronger for individuals with a more stable heart rate. Both heart-related effects were independent of alpha oscillations’ influence on somatosensory processing. We explain cardiac cycle timing effects in a predictive coding account and suggest that HEP-related effects might reflect spontaneous shifts between interoception and exteroception or modulations of general attentional resources. Thus, our results provide a general conceptual framework to explain how internal signals can be integrated into our conscious perception of the world.

## Significance

Our brain continuously receives signals from the body and the environment. Although we are mostly unaware of internal bodily processes, such as our heartbeats, they can affect our perception. Here, we show two distinct ways in which the heartbeat modulates conscious perception. First, increased heartbeat-evoked neural activity before stimulation is followed by decreased somatosensory detection. This effect can be explained by subjects adopting a more conservative decision criterion, which is accompanied by changes in early and late somatosensory-evoked responses. Second, stimulus timing during the cardiac cycle affects sensitivity but not criterion for somatosensory stimuli, which is reflected only in late somatosensory-evoked responses. We propose that these heartbeat-related modulations are connected to fluctuations of interoceptive attention and (unconscious) predictive coding mechanisms.

The neural response to an external stimulus and its access to consciousness depend on stimulus features as well as the state of the brain (1–5). Interestingly, functional states of other bodily organs, such as the heart, can also influence the perception of external stimuli. For example, several studies have reported that timing along the cardiac cycle (e.g., systole vs. diastole) impacts the perception of visual or auditory stimuli (refs. 6 and 7, but also see refs. 8 and 9 for nonsignificant heart phase-dependent effects). For the somatosensory system, we recently showed increased detection during diastole (10) similar to the other sensory domains (6, 7). Interestingly, a previous study had reported lower somatosensory sensibility during diastole (11) when stimulus presentation was at fixed time points during the cardiac cycle. Similar to perception, neural responses to visual and auditory stimuli are modulated across the cardiac cycle (12, 13). Most often they have been reported to be higher during diastole than systole (12, 13). A recent study (14) has also associated fluctuations of the heartbeat-evoked potential (HEP; refs. 15–17) with conscious detection of a visual stimulus.

While thus increasing evidence indicates that events related to cardiac function may modulate conscious perception, fundamental questions remain unanswered. Is it perceptual discrimination ability, that is, sensitivity in signal detection theory (SDT; ref. 18), that is influenced by cardiac activity? Or, might a bias to report the presence or absence of a stimulus underlie the effect, that is, criterion, in SDT? Are criterion-free decisions also affected by the heart? How are these perceptual effects reflected in evoked neural activity? More specifically, do these effects influence early, preconscious, somatosensory-evoked potentials (SEPs) or only the late components? Ultimately, how cardiac-related modulation of perceptual awareness relates to primary determinants of sensory perception and evoked brain activity, such as prediction, attention, and background neural activity, is unknown.

The current study targets mechanisms linking heart, brain, and perception using a somatosensory detection and localization task with electroencephalography (EEG) recordings. In an SDT-based design, we identify differential effects of two heartbeat-related phenomena: 1) stimulus timing during the cardiac cycle and 2) the amplitude of the HEP on somatosensory perception and evoked potentials. We argue that these findings are in line with a predictive coding account for cardiac phase-related sensory fluctuations and likely to be related to spontaneous shifts between interoception and exteroception as indexed by the HEP amplitude.

## Results

**Fig. 1.**
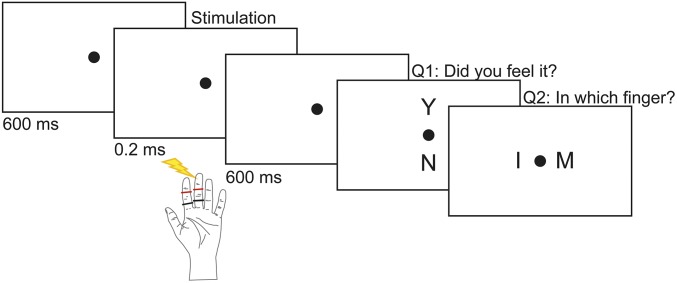
Experimental paradigm. Thirty-seven subjects received a weak electrical pulse to the left index or the middle finger in 800 out of 960 trials over eight experimental blocks. Subjects were told that every trial contained a stimulus; however, in 160 pseudorandomized trials no stimulus was actually presented. In every trial, participants were asked to first perform a yes/no detection task and then a location discrimination task.

Thirty-seven participants were presented weak somatosensory (electrical) stimuli to either the left index or middle finger in a combined yes/no detection and location discrimination task (Fig. 1). Both EEG and electrocardiography (ECG) were recorded. On average, participants detected 51.0 ± 10.5% (mean ± SD) of the somatosensory stimuli with a false alarm rate of 8.4 ± 7.7%. This corresponds to a mean detection sensitivity, *d′*, of 1.57 ± 0.57 and a decision criterion, *c*, of 0.76 ± 0.32. Participants correctly localized 73.3 ± 6.6% of stimuli (fingerwise), corresponding to a mean localization sensitivity of 0.90 ± 0.32. Participants correctly localized 88.9 ± 7.9% of hits and 57.0 ± 6.9% of misses.

### Detection Varies across the Cardiac Cycle.

**Fig. 2.**
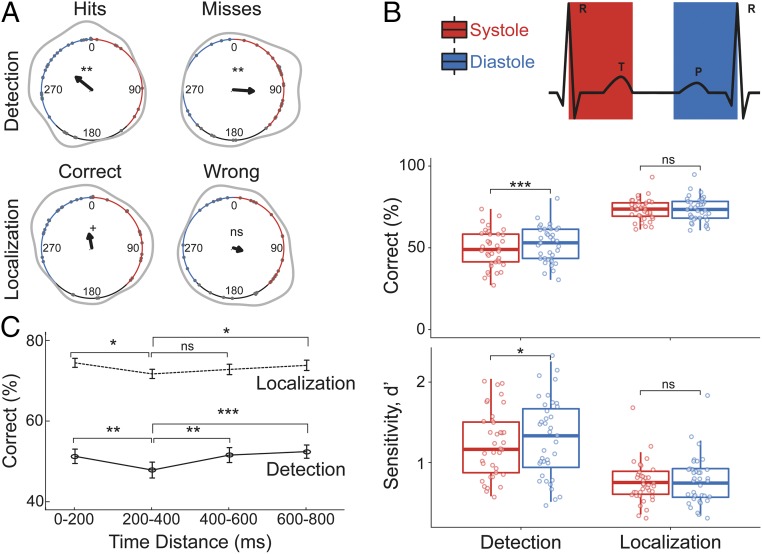
Conscious detection of somatosensory stimuli varies across the cardiac cycle. (*A*) Distribution of hits (*Top Left*), misses (*Top Right*), correct localizations (*Bottom Left*), and wrong localizations (*Bottom Right*) across the cardiac cycle (the interval between two R-peaks at 0/360°). Gray points show subjects’ mean degrees. The black arrows point toward the overall mean degree and its length indicates the coherence of individual means. The gray lines depict the circular density of individual means. The overall mean systole and diastole lengths are shown with red and blue, respectively. Hits and misses were nonuniformly distributed across the cardiac cycle (Rayleigh tests, $\bar{R}$ = 0.40, *P* = 0.003 and $\bar{R}$ = 0.40, *P* = 0.004, respectively). While correct localizations showed a trend toward a nonuniform distribution (*P* = 0.067), wrong localizations did not show a significant deviation from uniform distribution (*P* = 0.35). (*B*, *Top*) Correct detection and localization percentages during systole and diastole. Participants had more correct detections in diastole (*t*_36_ = −3.95, *P* = 3⋅10^−4^). No statistically significant difference between systole and diastole was found for correct localization (*P* = 0.54). (*B*, *Bottom*) Detection and localization sensitivity (*d′*) between systole and diastole. Detection sensitivity was significantly higher in diastole than systole (*t*_36_ = −2.38, *P* = 0.008), and localization sensitivity did not differ significantly between the two cardiac phases (*P* = 0.38). (*C*) Correct detection and localization of somatosensory stimuli relative to their distance from the previous R-peak. Both detection and localization performances were lowest 200 to 400 ms after the R-peak. (post hoc paired *t* test between 0 and 200 and 200 and 400 ms for detection: *t*_36_ = 3.76, *P* = 6⋅10^−4^ and localization: *t*_36_ = 2.88, *P* = 0.007). Error bars represent SEMs. ^+^*P* < 0.08, *P < 0.05, **P < 0.005, ***P < 0.0005; ns, not significant.

We hypothesized that hits were more likely to occur in a later phase of the cardiac cycle, whereas misses would occur in an earlier phase (10). We used three complementary approaches to test this hypothesis. First, we used circular statistics (19), which allows an assessment of the entire cardiac cycle, without distinguishing systole and diastole, whose relative lengths are differentially affected by changes in the duration of the cardiac cycle (see *Circular Analysis* for details). A Rayleigh test showed that hits were not uniformly distributed, $\bar{R}$ = 0.40, *P* = 0.003 (Fig. 2*A*), with a mean angle of 308.70° corresponding to the later cardiac cycle phase (i.e., diastole). Similarly, the distribution of misses was not uniform, $\bar{R}$ = 0.40, *P* = 0.004 (Fig. 2*A*), with a mean angle of 93.84°, located in the early phase of the cardiac cycle (i.e., systole). We observed a trend in the distribution of correct localizations toward the later phases of the cardiac cycle ($\bar{R}$ = 0.28, *P* = 0.067). The distribution of wrong localizations was not significantly different from a uniform distribution, $\bar{R}$ = 0.17, *P* = 0.35 (Fig. 2*A*).

### Detection Rate and Sensitivity Are Higher during Diastole Compared to Systole.

To account for the biphasic nature of the cardiac cycle, we also examined detection and localization performance by segmenting each cardiac cycle into systole and diastole: We operationalized the systolic time window for each cardiac cycle as the time between the R-peak and the end of the t-wave (see *Binary Analysis* for further details). Based on the duration of this systolic window, we defined a diastolic window of equal length at the end of each cardiac cycle (Fig. 2*B*). As suggested by our first analysis, the detection rate for the weak stimuli was significantly higher during diastole (mean [M] = 52.41%) than systole (M = 49.53%), *t*_36_ = −3.95, *P* = 3⋅10^−4^ (Fig. 2*B*). Increased detection rate during diastole was observed for 27 out of 37 participants. However, the false alarm rate did not differ significantly between systole (M = 8.50%) and diastole (M = 8.19%), *t*_36_ = 0.54, *P* = 0.59. There was no significant difference between stimulus intensities in systole and diastole (*t*_36_ = 0.57, *P* = 0.57; *SI Appendix*, Table S1). Additionally, we tested whether the latency to response differed between systole and diastole but did not find a significant difference (*t*_36_ = 0.83, *P* = 0.41).We furthermore tested whether the effect of cardiac phase on detection correlated with the heart rate or the heart rate variability (HRV, i.e., the SD of time duration between two successive R-peaks [RR intervals]) of individuals. While there was no significant correlation between subject’s heart rate and their detection rate variation between systole and diastole (Pearson’s correlation, *r* = 0.01, *P* = 0.95), subjects’ HRV negatively correlated with their detection rate difference (*r* = −0.36, *P* = 0.03; *SI Appendix*, Fig. S1).

SDT was applied to test whether the increased detection rates in diastole were due to increased perceptual sensitivity (*d′*) or due to adopting a more liberal response strategy (criterion). Detection sensitivity was significantly higher in diastole (M = 1.59) than systole (M = 1.48), *t*_36_ = −2.38, *P* = 0.008 (Fig. 2*B*). For the criterion, no significant difference between systole (M = 0.75) and diastole (M = 0.73) was found, *t*_36_ = 0.71, *P* = 0.48. Localization performance was also tested across the cardiac cycle. Correct localization rate did not differ significantly between systole (M = 73.27%) and diastole (M = 73.68%), *t*_36_ = −0.62, *P* = 0.54. Likewise, localization sensitivity was not significantly different between systole (M = 0.90) and diastole (M = 0.93), *t*_36_ = −0.89, *P* = 0.38 (Fig. 2*B*).

Finally, other heartbeat-associated physiological events (e.g., the pulse wave) are temporally coupled with the onset of systole. Therefore, in an exploratory analysis we assessed the effect of the absolute time delay of somatosensory stimulation from the previous R-peak on detection and localization rates. Detection and localization rates were significantly different between four time windows: 0 to 200, 200 to 400, 400 to 600, and 600 to 800 ms (within-subject ANOVA, *F*_3,108_ = 7.25, *P* = 2⋅10^−4^ and *F*_3,108_ = 3.97, *P* = 0.01). Detection and localization was lowest 200 to 400 ms after the R-peak (post hoc paired *t* test between 0- to 200- and 200- to 400-ms windows for detection: *t*_36_ = 3.76, *P* = 6⋅10^−4^ and localization: *t*_36_ = 2.88, *P* = 0.007; between 200 to 400 and 400 to 600 ms for detection: *t*_36_ = −3.61, *P* = 9⋅10^−4^ and localization: *t*_36_ = −1.36, *P* = 0.18; Fig. 2*C*). Significant differences were found for the sensitivity (main effect of time, *F*_3,108_ = 6.26, *P* = 6⋅10^−4^; post hoc paired *t* test between 0 to 200 and 200 to 400 ms, *t*_36_ = 2.83, *P* = 0.008 and between 200 to 400 and 400 to 600 ms, *t*_36_ =−3.48, *P* = 0.001) but not for the criterion (*F*_3,108_ = 0.10, *P* = 0.96; *SI Appendix*, Fig. S2).

### SEPs during Diastole Compared to Systole.

**Fig. 3.**
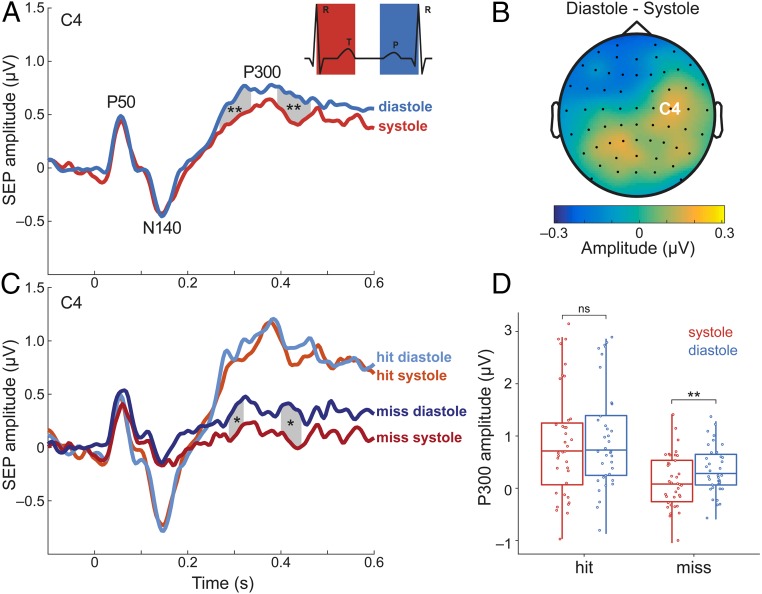
SEPs for stimulations during systole vs. diastole (*A*) The difference in P300 component of SEPs (electrode C4) between systole and diastole. SEPs were more positive for stimuli during diastole than systole between 268 to 340 ms and 392 to 468 ms after stimulus onset over contralateral somatosensory cortex (Monte Carlo *P* = 0.004 and *P* = 0.003, respectively, corrected for multiple comparisons in time). (*B*) The topography contrast between diastole and systole between 268 and 468 ms. The position of electrode C4 is shown on the head model. (*C*) SEPs for hits (lighter colors) and misses (darker colors) during systole (red) and diastole (blue). SEPs showed higher positivity for misses during diastole than during systole in two time windows: 288 to 324 ms and 400 to 448 ms (*P* = 0.02 and *P* = 0.01, respectively). (*D*) The mean SEP amplitude between 268 to 468 ms for detection and cardiac phases. *P < 0.05, **P < 0.005; ns, not significant.

Conscious somatosensory perception is known to correlate with greater amplitude of certain SEP components such as N140 and P300 (20). In line with the changes in somatosensory perception, we expected to find differences in SEPs during diastole compared to systole. We systematically compared SEPs during systole and diastole in the time window of 0 (stimulation onset) to 600 ms with a cluster-based permutation *t* test. SEPs over the contralateral somatosensory cortex (indexed by C4 electrode) showed greater positivity when stimulation was performed during diastole than systole in two temporal clusters: 268 to 340 ms and 392 to 468 ms (Monte Carlo *P* = 0.004 and *P* = 0.003, respectively, corrected for multiple comparisons in time; Fig. 3*A*). SEPs for hits during diastole and systole did not differ significantly (smallest Monte Carlo *P* = 0.27). SEPs for misses, however, differed between systole and diastole over the contralateral somatosensory area. Higher positivity was observed in diastole compared to systole in time windows of 288 to 324 ms and 400 to 448 ms, respectively (Monte Carlo *P* = 0.02 and Monte Carlo *P* = 0.01, respectively; Fig. 3*C*).

We used a within-subject ANOVA with the factors detection (hit vs. miss) and cardiac phase (systole vs. diastole) to examine their effect on the P300 component of the SEPs. The P300 latency was determined in the 268- to 468-ms interval by merging the two time clusters observed for SEP differences between systole and diastole. We found significant main effects of detection (*F*_1,36_ = 33.29, *P* = 1⋅10^−6^) and cardiac phase (*F*_1,36_ = 8.26, *P* = 0.007). We did not observe a significant interaction effect (*F*_1,36_ = 2.55, *P* = 0.12).

To ascertain that the SEP differences during systole and diastole originate from somatosensory cortex, a source reconstruction was performed (see *SI Appendix*, *Methods* for details). On source level, we confirmed the significant difference in P300 amplitude during systole and diastole in the contralateral somatosensory cortex (*t*_36_ = −2.55, *P* = 0.01; *SI Appendix*, Fig. S3). In exploratory analyses, we tested SEPs in other brain areas known to influence heart–brain interactions and SEP amplitudes: right anterior insula (21), right inferior parietal lobule (rIPL; ref. 14), bilateral anterior and posterior cingulate (ACC and PCC; refs. 14 and 22) as well as bilateral lateral prefrontal cortices (LPFC; ref. 22). We did not find significant differences in the SEPs between systole and diastole in these regions (*SI Appendix*, Table S2).

### HEPs Predict Somatosensory Detection.

**Fig. 4.**
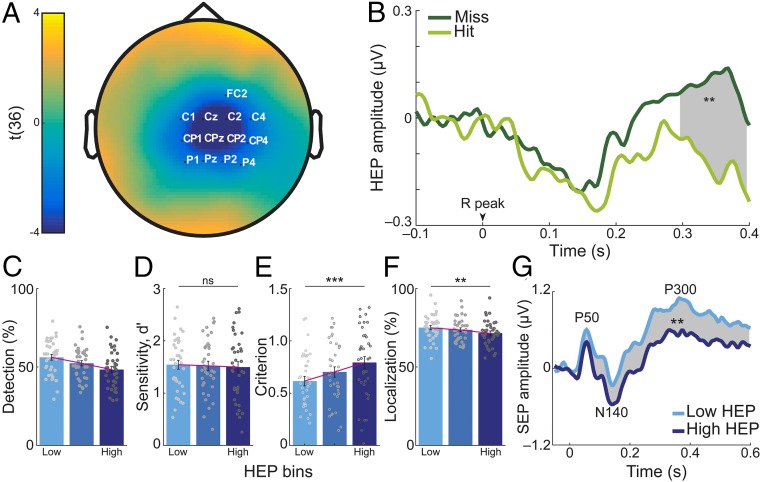
HEPs before stimulus onset predicted somatosensory detection. (*A*) Topographical map of *t* values for HEP differences preceding hits and misses: Grand average across 37 participants in the 296- to 400-ms time window, where a significant difference (misses > hits) was observed on the highlighted electrodes (Monte Carlo *P* = 0.004 corrected for multiple comparisons in time and space). (*B*) Prestimulus HEPs averaged across the cluster. (*C*–*F*) Single-trials were sorted according to the mean HEP amplitude (across the cluster in the 296- to 400-ms time window) and split into three equal bins for each subject. (*C*) As the HEP amplitude increased, the detection rate decreased. (*D*) This decrease was not associated with a significant change in detection sensitivity (*P* = 0.84), (*E*) but correlated with an increase in criterion, that is, reporting stimulus presence less often regardless of actual stimulus presence (*P* < 0.0005). (*F*) Similar to the decrease in detection rate, correct localization rate decreased with increasing HEP amplitude (*P* = 0.003). The gray points on the bar plots represent individual subjects. (*G*) SEP amplitudes for trials in the low and high HEP bins. A significant difference in SEP amplitudes for the low and high HEP bin was observed between 32 and 600 ms poststimulation at contralateral somatosensory cortex (C4 electrode; Monte Carlo *P* = 0.004 corrected for multiple comparisons in time). Error bars represent SEMs. **P < 0.005, ***P < 0.0005; ns, not significant.

HEPs are cortical electrophysiological responses time-locked to the R-peak of the ECG and are thought to represent neural processing of cardiac activity (15, 23, 24). We tested whether HEPs immediately preceding stimulus onset predicted somatosensory detection. To ensure that the time window for the HEP, 250 to 400 ms after the R-peak (15, 23, 24), was free of neural responses to the stimulation, we only included trials where the stimulus occurred at least 400 ms after the preceding R-peak (i.e., during diastole). We averaged the EEG data locked to the R-peak separately for hits and misses and submitted the 250- to 400-ms post R-peak time window to a cluster-based permutation *t* test. Prestimulus HEPs significantly differed between hits and misses over the contralateral somatosensory and central electrodes between 296 and 400 ms (Monte Carlo *P* = 0.004 corrected for multiple comparisons in space and time; Fig. 4 *A* and *B*) with a significantly higher positivity for misses. No significant changes were found in either heart rate or HRV between hits and misses included in the HEP analyses (*t*_36_ = 1.51, *P* = 0.14 and *t*_36_ = −0.61, *P* = 0.55, respectively). Therefore, the observed differences in HEPs cannot be attributed to changes in heart rate or HRV between hits and misses (14).

Subsequently, we calculated the prestimulus HEPs averaged across the cluster electrodes in the 296- to 400-ms time window separately for different detection responses (e.g., hits and misses). Similarly, we computed HEPs for cardiac cycles outside the stimulation window (Fig. 1). Nonstimulation-related HEPs showed significantly more positivity than those preceding hits (paired *t* test, *t*_36_ = 4.83, *P* = 3⋅10^−5^) and a trend toward more positivity compared to those preceding misses (paired *t* test, *t*_36_ = 1.90, *P* = 0.07). HEP amplitudes preceding correct rejections showed significantly less positivity than HEPs preceding hits (paired *t* test, *t*_36_ = 4.22, *P* = 2⋅10^−4^) and were not significantly different from HEPs preceding misses (paired *t* test, *t*_36_ = 1.63, *P* = 0.11).

Next, we tested whether the HEP amplitude difference between hits and misses reflected a change in sensitivity or criterion according to SDT (Fig. 4 *D* and *E*). We sorted single trials according to mean HEP amplitude (across the cluster electrodes in the 296- to 400-ms time window) and split them into three equal bins (the number of HEP bins was chosen for comparability with a previous study; ref. 12) for each participant. We found that detection rates decreased as the HEP amplitude increased. Since we already showed this effect in the cluster statistics, we did not apply any statistical test here to avoid “double dipping” (25). The decrease in detection rate with increasing HEP amplitude was associated with an increase in criterion. More specifically, participants were more conservative in their decision and reported detecting the stimulus less often, regardless of their actual presence, when HEP amplitude was higher (within-subject ANOVA, *F*_2,36_ = 10.30, *P* = 1⋅10^−4^). Simultaneously, their sensitivity did not change significantly (*F*_2,72_ = 0.17, *P* = 0.84). We then tested whether prestimulus HEP amplitude could also affect somatosensory localization. Increasing HEP levels were associated with decreases in localization rate (*F*_1.72,62.01_ = 10.27, *P* = 0.03; Fig. 4*F*). Correct localization of hits and misses did not significantly differ between HEP bins (*F*_2,72_ = 1.26, *P* = 0.29 and *F*_2,72_ = 0.28, *P* = 0.76; *SI Appendix*, Fig. S4), indicating that the change in localization rate, associated with HEP amplitude, was connected with the change in detection rate.

We also tested whether prestimulus HEP amplitudes were associated with changes in SEP amplitudes. We applied a cluster-based permutation *t* test in the time window of 0 to 600 ms (0 = stimulation onset) to compare SEPs following low and high HEP amplitudes. Between 32 ms and 600 ms SEPs over the contralateral somatosensory cortex had higher positivity when stimulation was preceded by low HEP compared to high HEP amplitudes (Monte Carlo *P* = 0.004 corrected for multiple comparisons in time; Fig. 4*G*). On the source level, we confirmed that the amplitude of the earliest SEP component (P50) was significantly different following low and high HEP amplitudes in the contralateral primary somatosensory cortex (*SI Appendix*, Fig. S5). In further exploratory analyses, we tested whether differences in the P50 component could be observed in other brain areas involved in heart–brain interactions (cf. the previous section). Following high and low HEP amplitudes, there was a significant difference of P50 amplitude (false discovery rate-corrected) in the right anterior insula (*t*_36_ = 3.23, *P* = 3·10^−3^), the left and right PCC (*t*_36_ = −4.55, *P* = 6·10^−5^ and *t*_36_=−3.39, *P* = 2·10^−3^), and the left and right LPFC (*t*_36_ = −3.80, *P* = 5·10^−4^ and *t*_36_ = −4.14, *P* = 2·10^−4^) but not in the rIPL and the bilateral ACC (*SI Appendix*, Table S3).

### Prestimulus Sensorimotor Alpha Rhythm Predicts Somatosensory Detection and Localization.

**Fig. 5.**
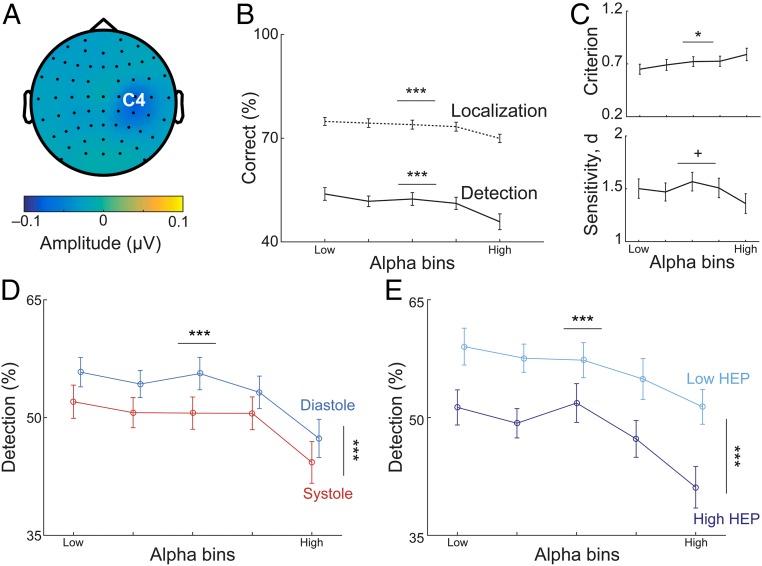
Prestimulus sensorimotor alpha amplitude affects somatosensory perception but does not mediate heartbeat-related perceptual effects. (*A*) Topography of prestimulus alpha (8 to 13 Hz) difference between hits and misses in the time window of 300 to 0 ms before stimulus onset. (*B*) Trials were sorted into five equal bins of increasing mean sensorimotor alpha amplitudes in the prestimulus time window of 300 to 0 ms over contralateral somatosensory cortex (C4 electrode). Correct detection and localization rates are given for each alpha bin. Both detection and localization decreased as alpha amplitude levels increased (*P* = 3⋅10^−7^ and *P* = 4⋅10^−5^). (*C*) The decrease in detection rates with increasing alpha amplitude levels was associated with a significant increase in criterion, that is, a higher bias to miss the target (*P* = 0.006; *Top*) and a trend toward lower sensitivity (*P* = 0.07; *Bottom*). (*D*) For each alpha bin, detection rates are given separately for systole and diastole. Cardiac phase and alpha levels affected detection rate in an additive fashion (within-subject ANOVA test, *F*_1,36_ = 15.82, *P* = 3⋅10^−4^ and *F*_2.93,105.30_ = 12.05, *P* = 1⋅10^−6^). (*E*) For each alpha bin, detection rates are given separately for the trials with highest and lowest HEP, respectively. Prestimulus HEP amplitudes across the time window 296 to 400 ms after the R-peak were categorized in three equal bins for each participant, and detection rates were determined separately for the lowest and highest HEP conditions within each alpha bin. Both prestimulus factors, that is, HEP amplitudes and alpha amplitudes, influenced detection rates independently (within-subject ANOVA *F*_1,36_ = 38.71, *P* = 4⋅10^−7^ and *F*_4,144_ = 10.37, *P* = 2⋅10^−7^). Error bars represent SEMs. ^+^*P* < 0.08, *P < 0.05, ***P < 0.0005.

Given that alpha rhythm is known to influence sensory processing (2, 26–29), we assessed its effect on perception in our study as well as its possible interaction with heartbeat-related effects. Therefore, we sorted and divided trials into five equal bins (the number of alpha bins were chosen to be consistent with previous studies; refs. 25 and 26), according to the mean sensorimotor alpha amplitude between 300 and 0 ms before stimulus onset. We then calculated the percentage of correct detection and localization responses for every bin. Correct detection and localization responses decreased with increasing levels of alpha amplitude (within-subject ANOVA, *F*_2.77,99.74_ = 8.88, *P* = 3⋅10^−7^ and *F*_3.30,118.81_ = 6.11, *P* = 4⋅10^−5^; Fig. 5*B*). With increasing prestimulus alpha amplitude, participants had a more conservative criterion (*F*_4,144_ = 3.77, *P* = 0.006; Fig. 5*C*). Sensitivity did not change significantly but showed a trend toward a decrease (*F*_4,14_ = 2.20, *P* = 0.07; Fig. 5*C*).

### Sensorimotor Alpha Does Not Mediate Cardiac Phase Effect on Detection.

Since prestimulus sensorimotor alpha amplitude modulated somatosensory perception, we hypothesized that alpha oscillations mediated the effect of cardiac phase on detection. To test this hypothesis, we calculated detection rates separately for systole and diastole trials within each alpha bin, where alpha amplitudes were comparable (*F*_1,36_ = 0.89, *P* = 0.35). Both cardiac phase and alpha levels significantly correlated with detection rate (within-subject ANOVA test, *F*_1,36_ = 15.82, *P* = 3⋅10^−4^ and *F*_2.93,105.30_ = 12.05, *P* = 1⋅10^−6^) but there was no significant interaction effect (*F*_4,144_ = 0.34, *P* = 0.85; Fig. 5*D*). This result indicated that detection rates differed between systole and diastole in the presence of comparable sensorimotor alpha amplitude levels. Further confirmation of this relationship by fitting general linear mixed-effects models (GLMM) at a single-trial level is shown in *SI Appendix*, *Methods* and Table S4).

### Prestimulus Sensorimotor Alpha Does Not Mediate the Effect of HEP on Detection.

To test whether prestimulus alpha amplitude mediated the relationship between HEP and detection, detection rates were calculated separately for low and high HEP levels within each alpha bin, where alpha amplitudes were similar between low and high HEP (*F*_1,36_ = 0.14, *P* = 0.71). A within-subject ANOVA showed significant main effects of both HEP (*F*_1,36_ = 38.71, *P* = 4⋅10^−7^) and alpha amplitude levels (*F*_4,144_ = 10.37, *P* = 2⋅10^−7^) for the detection rate with no significant interaction between them (*F*_4,144_ = 0.75, *P* = 0.56; Fig. 5*E*). This result shows that the HEP effect was additive to the effect of alpha levels on detection (see also *SI Appendix*, Table S5 for additional GLMM analyses).

### Controls for Volume Conduction Effect.

Moreover, we ascertained that the observed SEP differences between the two cardiac phases as well as the HEP effect on detection were not likely to be explained by differences in cardiac electrical activity, which might have caused differences in the EEG by volume conduction (14, 16, 30). First, we examined whether possible ECG artifacts were successfully eliminated during the calculation of SEP differences between systole and diastole (see *Materials and Methods* for further details and *SI Appendix*, Fig. S6 *A*–*C*): We tested whether the ECG waveform difference between the systole and diastole trials were canceled out after ECG artifact correction (*SI Appendix*, Fig. S6 *D*–*F*). The comparison between two residual ECG waveforms for systole and diastole trials revealed no significant difference (no clusters were found; *SI Appendix*, Fig. S6*F*). Thus, the observed differences in SEP amplitudes between systole and diastole cannot be attributed to differences in cardiac electrical activity. Second, we checked whether the response to heartbeats preceding hits and misses differed in the ECG data. The ECG data looked similar for hits and misses (*SI Appendix*, Fig. S7*A*). The cluster statistics on the ECG data 296 to 400 ms after the R-peak did not show any significant difference between hits and misses (no clusters were found; *SI Appendix*, Fig. S7*A*). Correcting the EEG data for the cardiac artifact using independent component analysis did not significantly change the results (*SI Appendix*, Fig. S7*B*). Therefore, HEP differences preceding hits and misses cannot be explained due to differences in cardiac electrical activity.

## Discussion

We show that the timing of a somatosensory stimulus, with respect to the cardiac cycle, along with the amplitude of the prestimulus HEP shape conscious perception and the SEP. More specifically, detection rates were higher during diastole than systole and inversely related to the amplitude of the preceding HEP. Differential psychophysical effects of cardiac phase and HEP were observed on sensitivity and criterion, respectively. Furthermore, the cardiac phase influenced only late components of the SEPs (P300), whereas the effects of HEP amplitude were observed in both early (starting with P50) and late SEP components. While prestimulus alpha power also influenced perception and somatosensory processing, its effect was independent of both heartbeat-related effects on conscious perception, that is, alpha power and heartbeat-related events had an additive impact on somatosensory perception.

Our first main finding, the modulation of perception and neural response along the cardiac cycle, seems best explained by periodical modulations of perception in a predictive coding framework, in which the brain is continuously producing and updating a model of sensory input. This model not only concerns exteroceptive stimuli but also interoceptive signals such as the heartbeat. Each heartbeat and its concomitant pulse wave lead to transient physiological changes in the entire body. These repeating cardiac fluctuations are treated as predictable events and attenuated by the brain to minimize the likelihood of mistaking these self-generated signals as external stimuli (31, 32).

Of relevance for our study, heartbeat-related pressure fluctuations are tightly coupled with the firing pattern of afferent neurons in the fingers (33). These neurons fire in response to the pressure wave that reaches its maximum after around 200 to 400 ms after the R-peak within systole (34). We postulate that the same top-down mechanism, which suppresses the perception of heartbeat-related firing changes in afferent finger neurons (33), also interferes with the perception of weak external stimuli to the fingers. This would only occur if presented during the same time period in systole—and more precisely between 200 and 400 ms after the R-peak. So, we propose that there is a prediction regarding heartbeat-/pulse wave-associated neural events which leads to the suppression of weak external somatosensory stimuli occurring in this time window. This effect reflected changes in sensitivity, that is, a weak input during systole is more likely to be regarded as pulse-associated “internal noise,” and thus the differentiation between the stimulation and “noise” becomes more difficult. This could also explain why localization becomes worse during systole. Interestingly, a recent modeling study suggested that predictive mechanisms leading to attenuated integration of weak and neutral exteroceptive input might give rise to higher uncertainty about environmental “risks,” which the organism would compensate for by increasing the expectation for detecting fear/threat in the environment (35). This may explain why the detection of fear/threat stimuli—in contrast to our neutral somatosensory stimuli—is enhanced during systole (36).

Furthermore, we show that perceptual suppression during systole was stronger in individuals who had less HRV. Whether this latter effect is related to a possibly more accurate (temporal) prediction of the next heartbeat or another physiological mechanism associated with HRV such as the vagal tone cannot be differentiated based on our data.

A reduction of the P300 amplitude accompanied the cardiac phase-associated modulation in somatosensory perception and sensitivity during systole compared to diastole. If a peripheral mechanism (e.g., less sensitivity of receptors of peripheral nerves) were to underlie the cardiac cycle effects on perception, it would yield already a difference in earlier SEP components. Interestingly, the P300 component has been regarded as an indicator of the “prediction error” (37) such that its amplitude is expected to reduce with a more precise prediction (via a smaller prediction error). Thus, the suppression of the P300 component during systole suggests that the pulse-synchronous peripheral neural activity (33) elicits a central prediction of this peripheral neural activity. The P300 component has been also suggested to be an indicator of conscious awareness (38, 39). Fittingly, the suppression of recurrent activity within the somatosensory network in the later stages of stimulus processing would be expected to reduce P300 amplitude (38–40). Taken together, the decreased P300 amplitude and lower sensitivity for somatosensory stimuli during systole might indicate a less efficient propagation of neuronal activity to higher processing levels (41). In the context of the global neural workspace theory (38), decreased sensitivity prevents “ignition” of conscious perception of a stimulus by interfering with its processing within the higher-order sensory cortices. This prevents the broadcasting of the stimulus and therefore conscious perception of it.

Our second main finding links HEP amplitudes to the processing of weak somatosensory stimuli. Specifically, we show that HEP in the time range of 296 to 400 ms showed higher positivity for misses than hits over centroparietal electrodes. That is, the amplitude (positivity) of HEP was inversely related to detection as well as stimulus localization. Although cardiac physiology is known to modulate HEP amplitudes (42, 43), we could not detect any changes in cardiovascular measures (heart rate and HRV) with respect to HEP. However, we cannot rule out a possible effect of cardiac physiology in HEP-related effects since we did not assess all cardiac-related measures such as cardiac output. In an SDT-based analysis, we have shown that the HEP effect was mainly related to changes in the criterion, in other words, with increasing HEP, participants adopted a more conservative bias for detection. A conservative bias has been shown to be associated with lower baseline firing rate across different brain regions, pushing neurons away from the threshold for “ignition” (41). Supporting this mechanism of criterion, that is, changing baseline firing rates in the brain, we found that the increasing prestimulus HEP amplitudes had a negative effect on the amplitude of both early (P50) and later SEP components (N140, P300). In other words, we interpret the changes in SEP amplitudes as reflecting changes in criterion.

Following different levels of HEP, the source-localized P50 amplitude was also different in contralateral somatosensory cortex, right insular cortex, LPFC, and PCC. Right anterior insula has been proposed as an integral hub to mediate internally and externally oriented attention (21) that can trigger attentional switches via its reciprocal connections with the lateral prefrontal cortex—an important region for attentional control similar to PCC (44). Similar modulation of early SEP components (P50) has previously been shown along with shifts of spatial attention (27, 45). Given that HEP amplitude has been found to be significantly higher during interoceptive compared to exteroceptive attention (46–48), we propose that the modulations of HEP amplitude reflect attentional shifts between external stimuli and internal bodily states. In line with this view, it has been suggested that the sudden “ignition” of a spontaneous internal activity can block external sensory processing (49). Similarly, heartbeat-related signals, which have been suggested to contribute to spontaneously active and self-directed states of consciousness (14), might prevent “ignition” of the upcoming somatosensory stimulus. Overall, the most plausible explanation for our findings seems to be that a shift from external to internal attention, reflected by HEP amplitude increases, interferes with conscious perception of external somatosensory stimuli by decreasing the baseline firing rates within the somatosensory network. We are, however, aware that this interpretation is not definitely proven, and there might be alternative explanations, for example a modulation of overall attentional resources.

In the visual domain, a recent study also proposed that HEPs can predict the detection of weak stimuli (14). Interestingly, Park et al. (14) reported that larger heart-evoked activity measured using magnetoencephalography was associated with better external perception, while we observed the opposite pattern. These differences might be due to the different sensory modalities tested, that is, the allocation of attentional resources to interoception may vary for the detection of somatosensory and visual stimuli. In this context, it is important to note that interoception—in addition to neurotransmission via viscerosensory afferents—might be partly mediated or accompanied by somatic neurotransmission. For example, somatosensory afferents from the skin have been shown to be involved in cardiac interoception (50). Another interoceptive process, most likely to be informed by changes in the skin, is breathing. A recent study showed that when attention was directed to breathing, the somatosensory cortex showed a higher, and the visual cortex a lower, coupling to the anterior insular cortex, a key area for interoception (51). This result implies that interoception might interact with visual and somatosensory cortices differently. Furthermore, the somatosensory cortex has been indicated as one of the sources of HEPs (15, 52) and as playing a substantial role for interoception (21, 50). Therefore, it seems plausible that heart-related processes in the interoceptive cortices, notably involving somatosensory but less so visual areas, may interfere differently with the processing of exteroceptive somatosensory and visual signals.

Our third main finding relates heartbeat-associated effects to ongoing neural activity. First, we attempted to confirm the influence of prestimulus sensorimotor alpha activity on somatosensory perception as shown in previous studies (28, 53, 54). We observed that during periods of weak prestimulus alpha amplitude detection rates increased, which reflected a more liberal detection criterion. This finding is consistent with studies in the visual (26) and somatosensory domain (54). Even though detection has already been associated with lower alpha levels (2, 28, 53), the relationship between somatosensory localization and alpha amplitudes—to the best of our knowledge—has not been reported so far. In the visual domain, when localization and detection tasks were tested with a block design, detection but not localization was shown to vary across alpha levels (26). For the somatosensory domain, we showed that not only detection rates but also localization rates increased with decreasing prestimulus alpha amplitudes. Given the effect of alpha on somatosensory perception, we tested whether sensorimotor alpha oscillations modulated the heartbeat-related effects on detection. Our analysis showed that neither of the two heartbeat-related effects on perception (i.e., the cardiac phase and the HEP amplitude) was mediated by prestimulus alpha amplitude, but rather both are independent and additive to the effect of prestimulus sensorimotor alpha amplitude.

Several pathways relating cardiac activity to the brain have been suggested. Most notably, baroreceptor activation might inform cortical regions about timing and strength of each heartbeat (55). Baroreceptors are maximally activated during systole and their stimulation has been suggested to reduce cortical excitability (56). Thus, the systolic activation of baroreceptors might inform predictive mechanisms in the brain concerning when to attenuate the processing of heartbeat-coupled signals. Other than through baroreceptors, cardiac signals might also reach the cortex through direct projections of cardiac afferent neurons to the brain (57) or via somatosensory afferents on the skin (50) as discussed above. While presently it is not clear which of these pathways is most relevant for heart–brain interactions, our results are consistent with the notion of the somatosensory cortex as an important relay center for cardiac input (15, 21, 50, 52). How this relay center modulates the relationship between interoception and exteroception is an interesting topic for future research.

In conclusion, timing of stimulation along the cardiac cycle and spontaneous fluctuations of HEP amplitudes modulate access of weak somatosensory stimuli to consciousness and induce differential effects on SEPs. We explain these fundamental heart–brain interactions within the framework of interoceptive predictive coding (stimulus timing) and spontaneous shifts between interoception and exteroception (HEP amplitudes). These findings on heartbeat-related perceptual effects might serve as an example how in general body–brain interactions can shape our cognition.

## Materials and Methods

### Participants.

Forty healthy volunteers were recruited from the database of the Max Planck Institute for Human Cognitive and Brain Sciences, Leipzig, Germany. Three subjects were excluded from the analysis due to technical problems during the experiment. Data from 37 subjects were analyzed (20 females, age: 25.7 ± 3.9 y [mean ± SD], range: 19 to 36 y). Some experimental blocks were excluded from the data analysis due to data acquisition failures (eight blocks from five subjects), false alarm rates >40% (eight blocks from eight subjects), responding with the wrong finger in the task (four blocks from three subjects), and observation of closed eyes during the task (three blocks from one subject). After these exclusions, a total of 274 experimental blocks with 32,880 trials in 37 subjects were analyzed. The study was approved by the Ethical Committee of the University of Leipzig’s Medical Faculty (no. 462-15-01062015). All subjects signed written informed consent and were paid for their participation.

### Somatosensory Stimulation and Task Design.

Electrical finger nerve stimulation was performed with a constant-current stimulator (DS5; Digitimer) using single square-wave pulses with a duration of 200 μs. Steel wire ring electrodes were placed on the middle (anode) and the proximal (cathode) phalanx of the index and the middle finger of the left hand, respectively.

In the experiment, participants performed a yes/no detection and a two-alternative forced-choice localization task on every trial. At the beginning of each trial, a black dot appeared on the screen for 600 ms. Participants then expected to get stimulation on either the index or the middle finger of their left hand. Six hundred milliseconds after the stimulation, participants “were asked” (via “yes/no?” on the screen) to report as quickly as possible whether they felt a stimulus on one of their fingers or not. They responded “yes” if they felt the stimulus and “no” if not by using their right index finger. Thereafter, participants were asked to answer where the stimulation has occurred. They were explicitly told “to guess” even if they reported not feeling the stimulus in the first question. If they located the stimulus on the left index finger, they were asked to use their right index finger to answer and to use their right middle finger if they located the stimulus on the left middle finger. The next trial started immediately after responding to the localization question. In total, every participant completed eight blocks. Each block contained 100 trials with electrical stimulation (50 trials for each finger) and 20 trials without any stimulation (catch trials). The duration of each block was ∼8 min. To find stimulus intensities with 50% detection probability (i.e., threshold), we applied a two-step procedure before starting the experiment. First, we roughly estimated the lowest stimulus intensity for which participants could report a sensation by applying the method of limits with ascending intensities separately for the index and the middle finger (27, 58). Second, we used a yes/no detection task (as described above) containing catch trials and six stimulus intensities around this predicted stimulus intensity (15% below, identical to, 20%, 40%, 60%, and 80% above) for each finger. The 50% threshold intensity for each finger was estimated from the participant’s psychometric function (59). To control for threshold stability, stimulus intensities were readjusted after each block.

Hit, miss, false alarm (FA), and correct rejection (CR) terms were calculated for the yes/no detection task in this study. A hit was reporting the presence of a stimulus when it was present; a miss was reporting the absence of a stimulus even though it was present. For catch trials (i.e., no stimulus was presented), an FA was reporting the presence of a stimulus, while a CR was reporting its absence. The terms “correct localization” and “wrong localization” were used to describe the localization task performance. Correct localization was reporting the stimulus location correctly; wrong localization was reporting it incorrectly.

### Recordings.

EEG was recorded from 62 scalp positions distributed over both hemispheres according to the international 10–10 system, using a commercial EEG acquisition system (actiCap, BrainAmp; Brain Products). The midfrontal electrode (FCz) was used as the reference and an electrode placed on the sternum as the ground. Electrode impedance was kept ≤5 kΩ for all channels. EEG was recorded with a bandpass filter between 0.015 Hz and 1 kHz and digitized with a sampling rate of 2.5 kHz. An ECG electrode connected to the EEG system was placed under the participant’s left breast to record the heart activity.

### Data Analysis.

We applied two complementary approaches—circular and binary analysis—to examine detection and localization across the cardiac cycle (60). For these analyses, we first extracted the R-peaks from the ECG data by using Kubios HRV Analysis Software 2.2 (The Biomedical Signal and Medical Imaging Analysis Group, Department of Applied Physics, University of Kuopio, Finland) and visually corrected for inaccurately determined R-peaks (<0.1%). From RR interval time series during the whole experiment, we calculated the SD of RR intervals (SDNN) and natural-log transformed SDNN values to calculate HRV (61, 62).

### Circular Analysis.

We tested detection and localization over the entire cardiac cycle, from one R-peak to the next one, by using circular statistics, which corrects for different durations of the cardiac cycle both inter- and intraindividually and accounts for its oscillatory nature (19). We calculated the relative position of the stimulus onset within the cardiac cycle with the following formula:

[(onset time–previous R-peak time)/(subsequent R−peak time–previous R−peak time)] × 360,

which resulted in values between 0° and 360° (0 indicating the R-peak before stimulus onset). The distribution of stimulus onsets was tested individually for each participant with a Rayleigh test for uniformity. Two participants were excluded from further circular analyses due to nonuniformly distributed stimulation onsets across the cardiac cycle ($\bar{R}$ = 0.06, *P* = 0.04; $\bar{R}$ = 0.06, *P* = 0.03). For the rest of the participants (*n* = 35), the assumption of uniform onset distributions was fulfilled. We calculated the mean phase value at which different performances occurred (detection task: hit and miss; localization task: correct localization and wrong localization) for each participant. At the group level, it was tested whether the distribution of a specific performance score (e.g., hits) deviated from the uniform distribution with Rayleigh tests (19). The Rayleigh test depends on the mean vector length out of a sample of circular data points and calculates the mean concentration of these phase values around the circle. A statistically significant Rayleigh test result indicates the nonuniform distribution of data around the circle, that is, the cardiac cycle.

### Binary Analysis.

Considering the biphasic nature of cardiac activity, detection and localization performances were compared between the systolic and diastolic phases of the cardiac cycle. We defined systole as the time between the R-peak and the end of the t-wave (10). We used the systolic length of each cardiac cycle to define diastole as a diastolic window of equal length placed at the end of the cardiac cycle. The equal length of systole and diastole was used to equate the probability of having a stimulus onset in the two phases of the cardiac cycle. To determine the end of t-wave, a trapezoidal area algorithm was applied in each trial (63). This method has advantages compared to an approach with fixed bins (e.g., defining systole as the 300-ms time window following the R-peak) because it accounts for within- and between-subject variations in the length of systole and diastole (i.e., the heart rate). The results of the automated algorithm were visually quality-controlled. Twenty-seven trials for which the algorithm failed to calculate t-wave end and produced an abnormal systole length (more than 4 SDs above or below the participant-specific mean systole) were removed from further binary analyses. Mean systole (and diastole) length obtained from these analyses was 333 ± 21 ms. Each trial was categorized depending on whether the stimulus occurred during systole or diastole. The average number of trials categorized as systole was 338 ± 51 and as diastole was 342 ± 59.

### Data Preprocessing.

EEG and ECG data were analyzed offline using EEGLAB (64) and FieldTrip (65) toolbox algorithms as well as custom-built scripts on a MATLAB platform (MathWorks Inc.). An antialiasing filter with a 112.5-Hz cutoff was used before down-sampling individual datasets to 250 Hz. After all blocks were concatenated, data were first high-pass-filtered with 0.5 Hz and then low-pass-filtered with 45 Hz using a fourth order of Butterworth filter. The EEG channels that had a flat line longer than 5 s or showed less than 85% correlation with its reconstructed activity from other channels were removed and interpolated using their neighboring channels. After a principal component analysis was applied, data underwent an independent component analysis (ICA) using an extended infomax algorithm to remove sources of heartbeat, ocular and muscle artifacts (66). ICA components with cardiac field artifact were determined by segmenting ICA components depending on the R-peak of the ECG electrode and visually selecting the components whose activities were matching the time course of R-peak and t-wave of the ECG. After removing artifactual ICA components, the artifact-free components were forward-projected for the subsequent analysis steps. Afterward, the data were rereferenced to the average reference.

### SEP.

Data were segmented from −1,000 to 2,000 ms with respect to stimulus onset separately for trials where the stimulation occurred during systole vs. diastole. After segmenting data, we performed baseline correction using 100- to 0-ms prestimulus window. Testing for the maximum positive deflection of the early SEP component P50 (40 to 60 ms) showed that the right primary somatosensory area, contralateral to the stimulated hand (67), was represented by the C4 electrode. Therefore, the statistical analysis of SEP amplitude was performed on the C4 electrode (68). To cancel out possible effects of blood circulation, we estimated the cardiac artifact in the EEG data. For this purpose, random triggers were placed over cardiac cycles outside the stimulation window (Fig. 1). Then, we classified the arbitrary triggers as systole or diastole depending on the position of the trigger in the cardiac cycle. After the classification, data were segmented around these triggers (−1,000 to 2,000 ms) and averaged separately for systole and diastole to estimate the cardiac artifact during systole and diastole for each EEG channel per subject. We baseline-corrected these signals 100 ms before the onset of the arbitrary triggers (*SI Appendix*, Fig. S7). To prevent any possible ECG-induced artifact on the SEPs, we subtracted the mean systolic and diastolic artifacts from the SEPs during systole and diastole trials, respectively (30).

### HEPs.

After preprocessing data as described above, we selected the cardiac cycles containing a stimulus. We only chose the trials in which the stimulus onset was at least 400 ms after the preceding R-peak (corresponding to diastole). We determined HEPs by segmenting the preprocessed EEG data from −1,000 to 2,000 ms around the R-peak separately for hits and misses as well as for correct localizations and wrong localizations. In this way, we could calculate the prestimulus HEPs, which have been reported between 250 and 400 ms after the R-peak (15, 23, 24).

### Time-Frequency Analyses.

We performed time-frequency analyses to investigate sensorimotor alpha activity locked to stimulus onset. For sensorimotor alpha, we selected ICA components representing sensorimotor rhythms to eliminate effects of the occipital alpha activity as described previously by our group (27, 68). One to seven components per participant (mean 3 ± 1 SD) were selected and included in the analysis of somatosensory oscillatory activity. We ensured that our selection of sensorimotor components corresponded to a source in primary somatosensory and motor areas in source level (see *SI Appendix*, Fig. S8 for details). Then, data were segmented (−1,000 to 2,000 ms) and ECG-induced artifacts for systole and diastole were calculated and subtracted from the data as described in the previous section. Morlet wavelet analysis was performed on every trial for frequencies from 5 to 40 Hz with number of cycles increasing linearly from 4 to 10. Thus, a wavelet at 10 Hz was 4.9 cycles long and had a temporal resolution of 0.10 s and a spectral resolution of 4.85 Hz. We focused on the effects of prestimulus alpha activity in our statistical analysis to test whether the perceptual effect of the cardiac cycle on detection is influenced by prestimulus oscillatory activity (−300 to 0 ms) over contralateral somatosensory area.

### Analyses according to SDT.

Sensitivity (*d′*) and criterion (*c*, response bias) were calculated according to SDT (69): *d′* and *c* were calculated as z(HR) − z(FAR) and −[z(HR) + z(FAR)]/2, respectively, with HR corresponding to hit rate and FAR corresponding to false alarm rate. A log-linear correction was used to compensate for extreme false alarm proportions (70) since 2 of the 37 participants produced no false alarms. Localization *d′* prime was calculated as √2 * z(correct localization rate).

### Statistical Analyses.

Assessment of statistical significance for “two-condition comparisons” in EEG data were based on cluster-based permutation *t* tests as implemented in the FieldTrip toolbox (65, 71). In this procedure, adjacent spatiotemporal or spatiospectrotemporal points for which *t* values exceed a threshold are clustered (cluster threshold *P* value: 0.05). Then the cluster statistics are calculated by taking the sum of *t* values of all points within each cluster. The type I error rate was controlled by evaluating the cluster-level statistics under a randomized null distribution of the maximum cluster-level statistics. To determine the distribution of maximal cluster-level statistics obtained by chance, condition labels were randomly shuffled 1,000 times. For each of these randomizations, cluster-level statistics were computed and the largest cluster-level statistic was entered into the null distribution. Finally, the experimentally observed cluster-level statistic was compared against the null distribution. Clusters with a *P* value below 0.05 (two-tailed) were considered “significant.” We expected to observe differences in SEPs over contralateral somatosensory cortex indexed by C4 electrode. Therefore, in the comparisons of somatosensory related activity, we only used cluster statistics to test whether two experimental conditions differed in time over contralateral somatosensory cortex. In contrast, we did not a priori define a spatial region for HEP analyses but expected to observe a HEP between 250 and 400 ms after the R-peak (15, 23, 24).

If the sphericity assumption was violated in within-subject ANOVA, Greenhouse–Geisser correction was applied. All statistical tests were two-sided.

### Data and Code Availability.

The consent forms signed by participants do not allow us to give free access to data but require us to check that data are shared with members of the scientific community. Therefore, we stored data and code in the Open Science Framework and will make the link available upon request to researchers.

## Supplementary Material

Supplementary File
