# Supplementary material for: Heart–brain interactions shape somatosensory perception and evoked potentials
Source: Proc Natl Acad Sci U S A. 2020 Apr 27;117(19):10575–84. doi: 10.1073/pnas.1915629117 (PMC7229654; doi:10.1073/pnas.1915629117)
Supplement: Supplementary File [file pnas.1915629117.sapp.pdf]

PNAS
www.pnas.org

Supplementary Information for

Heart-Brain Interactions Shape Somatosensory Perception and Evoked Potentials

Esra Al, Fivos Iliopoulos, Norman Forschack, Till Nierhaus, Martin Grund,
Paweł Motyka, Michael Gaebler, Vadim V. Nikulin, Arno Villringer

Corresponding authors: Esra Al and Arno Villringer
Emails: esraal@cbs.mpg.de and villringer@cbs.mpg.de

This PDF file includes:

Supplementary text
Figures S1 to S8
Tables S1 to S5
SI References

www.pnas.org/cgi/doi/10.1073/pnas.1915629117

Supplementary Information Text

Methods

Source reconstruction. Source localization was performed with the BrainStorm toolbox (1) using individually measured electrode positions. When available, individual brain anatomies (19 subjects) and otherwise a template brain anatomy (ICBM152; 2) were used. Cortical surfaces were segmented from structural MRI data (MPRAGE) using Freesurfer (3) and a 3-shell boundary element model (BEM) was constructed to calculate the lead field matrix with OpenMEEG (4). We used eLORETA to compute orientation-constrained sources for each condition and subject (5). The MATLAB code for eLORETA algorithm is available in the MEG/EEG Toolbox of Hamburg (METH; https://www.uke.de/english/departments-institutes/institutes/neurophysiology-and-pathophysiology/research/research-groups/index.html). Individual source data were then projected to the ICBM152 template (2). Cortical anatomy was segmented according to Destrieux atlas (6).

General linear mixed-effects modeling (GLMM). This method was used for mediation analyses since they both acknowledge both between- and within-participant variations in the data from the model's fixed-effect estimates. GLMM was conducted in R (R Core Team, 2014) within the lme4 framework (7). The models were defined in the following form: outcome ~ predictor(s) + (predictor(s) | subject), which fits predictors of the fixed effect part (next to the "~") and predictors of the random effects part (in brackets) grouped by a factor, for which the predictors vary randomly, in our case, subjects.

First, we used GLMM to test whether the cardiac phase effect on detection was mediated by the prestimulus alpha amplitude. We computed five GLMMs regressing detection outcome (hit or miss): (1) one null model assuming no relationship, i.e., only the intercept served as predictor; (2,3) two models including either cardiac phase or alpha amplitudes as the fixed and random effect to regress detection; (4) an additive model regressing detection on both cardiac phase and alpha amplitude and (5) an interactive model assuming an interaction between cardiac phase and alpha amplitude to regress detection (see **Table S4**). Second, we used GLMM to test whether prestimulus alpha mediated the effect of HEP on detection. We computed five GLMMs regressing detection outcome: (6) one null model; (7,8) two models including either HEP or alpha amplitudes as the fixed and random effect to regress detection; (9) an additive model regressing detection on both HEP and (10) alpha amplitude and an interactive model assuming an interaction between these two predictors (see **Table S5**). In all the models containing alpha as a predictor, we used natural logarithmic transformation of alpha amplitude to normalize its distribution. To determine the best GLMM model explaining the data, maximum-likelihood ratio test statistics, which account for model complexity, were used.

Supplementary GLMM Results

To test the relationship between prestimulus sensorimotor alpha amplitude, cardiac phase and detection, general linear mixed-effects modeling (GLMM) regressions were fitted at the single-trial level. Regressions that included only the cardiac phase or only the alpha amplitude were highly significant compared to a null model, i.e., a model with no relationship assumed (*cardiac model*: $\chi^2 = 18.07$, $p = 4{\cdot}10^{-4}$; *$alpha_1$ model*: $\chi^2 = 121.71$, $p = 2{\cdot}10^{-16}$; **Table S4**). The comparison of the *$alpha_1$ model* and the *cardiac model* favored the *$alpha_1$ model* ($\chi^2 = 103.64$, $p = 2{\cdot}10^{-16}$; **Table S4**). The *$additive_1$ model* that included both cardiac phase and alpha amplitude fitted the data significantly better than the *$alpha_1$ model* ($\chi^2 = 17.41$, $p = 0.002$) and an *$interaction_1$ model* that included an interaction between cardiac phase and alpha ($\chi^2 = 1.51$, $p = 0.91$; **Table S4**). To illustrate the best model, the *$additive_1$ model,* with numbers: If a stimulus was preceded by an alpha amplitude of 0.5 µV (1 standard deviation below the mean amplitude), the detection rates for stimuli in diastole and systole would be 56% and 53%, respectively. When prestimulus alpha amplitude increased to 1.4 µV (1 standard deviation above the mean amplitude), the detection rates for stimuli in diastole and systole would decrease to 49% and 46%, respectively. In summary, these results suggest that sensorimotor alpha and cardiac phase have independent effects on detection, i.e., alpha is not mediating the effect of cardiac phase on somatosensory detection.

Similarly, to confirm the additive effect of the amplitudes of prestimulus sensorimotor alpha and HEP on detection at the single-trial level, we calculated GLMM regression fits (cf. previous section). Regressions that included only alpha or only HEP, respectively, as predictors were highly significant compared with a null model (*alpha₂ model*: ($\chi^2 = 60.27$, $p = 5{\cdot}10^{-13}$; HEP model: $\chi^2 = 85.29$, $p = 2{\cdot}10^{-16}$; **Table S5**). The comparison of the *alpha₂ model* and the *HEP model* favored the *HEP model* ($\chi^2 = 25.02$, $p = 2{\cdot}10^{-16}$; **Table S5**). The *additive₂ model* including both HEP and alpha amplitude in the regression fitted the data better than the *alpha₂ model* ($\chi^2 = 62.73$, $p = 1{\cdot}10^{-12}$) and the *interaction₂ model* ($\chi^2 = 0.57$, $p = 0.45$; **Table S5**). To illustrate the best model, the *additive₂ model,* with numbers: If a stimulus was preceded by a HEP amplitude of -1.7µV and an alpha amplitude of 0.5µV (1 standard deviation below the mean amplitude), the probability of detecting a stimulus was 59%. This probability would decrease to 51% if only the HEP amplitude would increase to 1.6µV and to 51% if only the alpha amplitude would increase to 1.4µV. If both HEP and alpha amplitudes would increase to 1.6µV and 1.4µV (one standard deviation above the mean amplitude), respectively, the detection probability would decrease to 43%. The GLMM results further support that sensorimotor alpha and HEP have independent effects on detection. Thus, alpha is also not mediating the effect of HEP on somatosensory detection.

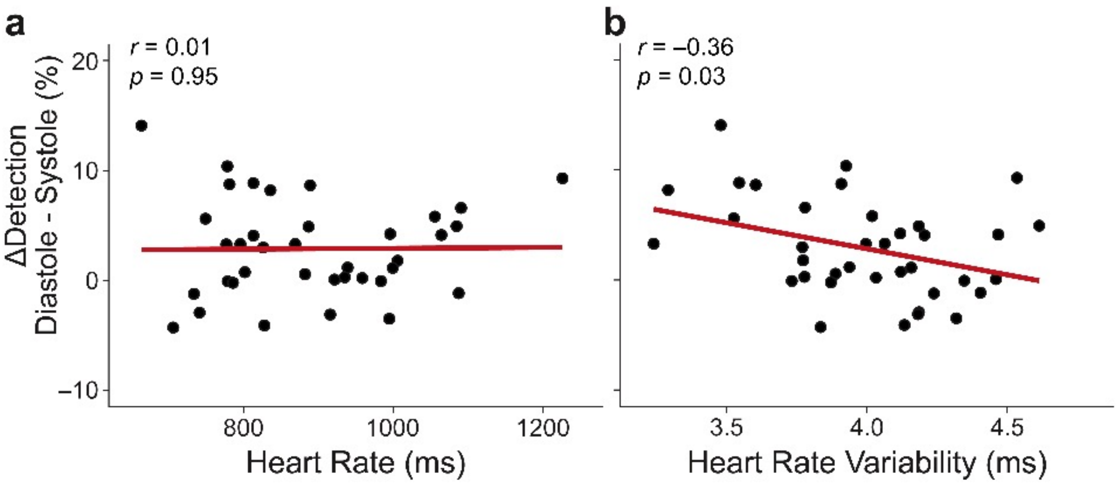

Figure S1. The change of detection between diastole and systole and its correlation with heart rate and heart rate variability. (a) Heart rate of subjects did not significantly correlate with their detection performance change between diastole and systole (Pearson's correlation $r = 0.01$, $p = 0.95$) (b) Heart rate variability (i.e., the standard deviation of RR intervals, SDNN) of subjects negatively correlated with the change of detection performance between systole and diastole ($r = -0.36$, $p = 0.03$).

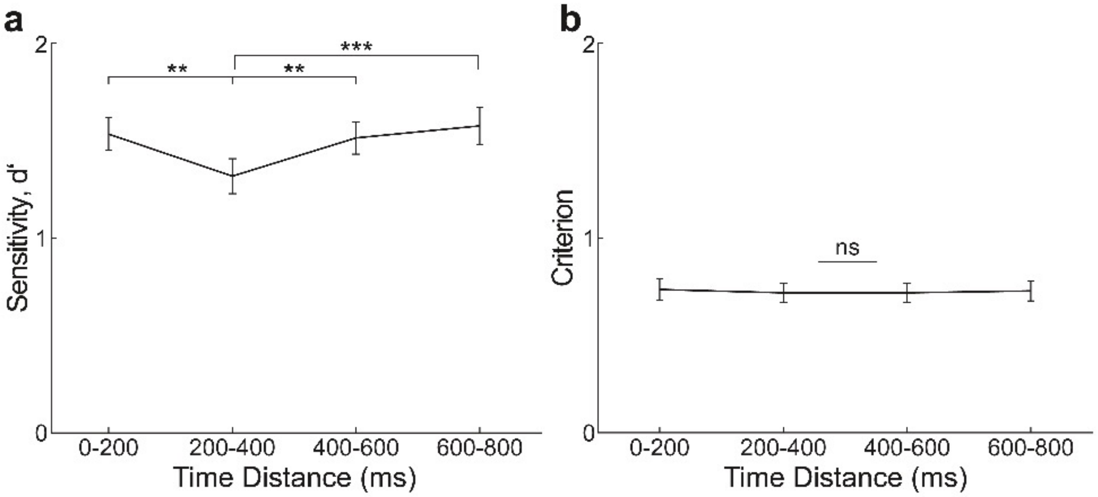

Figure S2. Sensitivity and criterion across four time windows of stimulus onset relative to the previous heartbeat (R-peak). (a) The detection sensitivity (d') was lowest 200 – 400 ms after the R-peak (*post-hoc* paired *t*-test between 0 – 200 and 200 – 400 ms, $t_{36} = 2.83$, $p = 0.008$ and between 200 – 400 and 400 – 600 ms, $t_{36} = -3.48$, $p = 0.001$) (b) Criterion did not differ significantly between the four time windows (main effect of time, $F_{3,108} = 0.10$, $p = 0.96$). Error bars represent SEMs. **$p < 0.005$, ***$p < 0.0005$. ns, not significant.

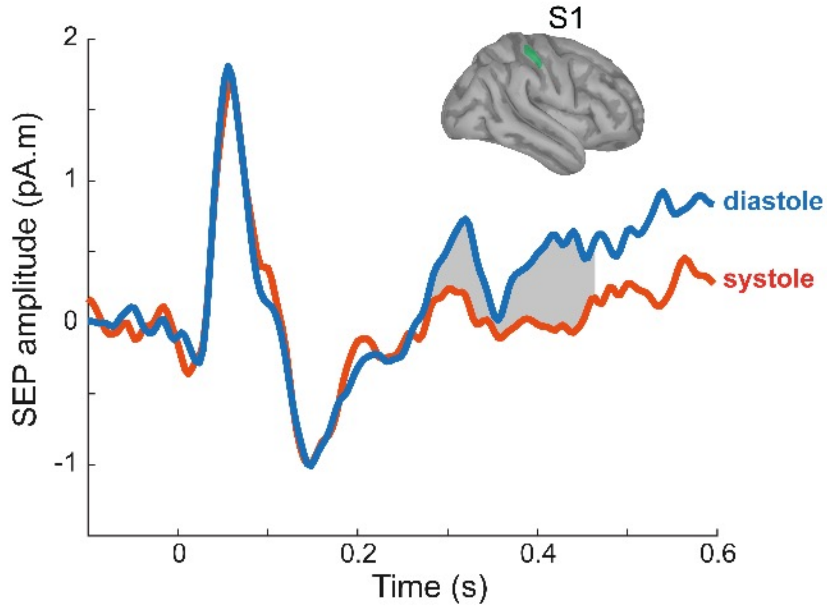

Figure S3. Somatosensory-evoked potentials (SEPs) for stimulations during systole versus diastole in source level. The source-reconstructed P300 amplitude was significantly different between systole and diastole in contralateral somatosensory cortex (S1) similar to the sensory data (t_{36} = -2.55, p = 0.01).

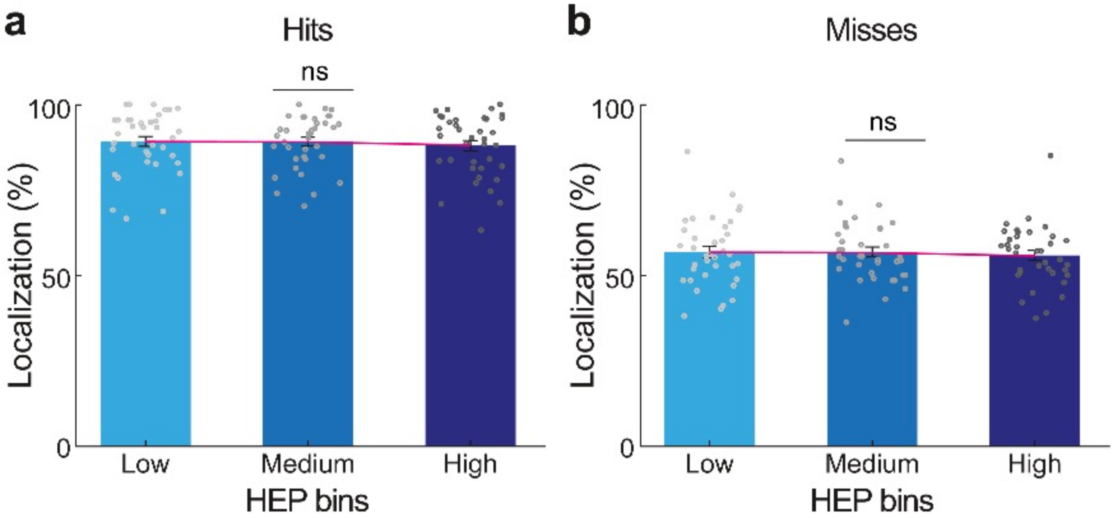

Figure S4. Correct localization of hits and misses across heartbeat-evoked potential (HEP) bins. (**a**) Correct localization of hits did not significantly change across increasing levels of HEP (within-subject ANOVA, $F_{2,72} = 1.26$, $p = 0.29$) **(b).** Correct localization of misses did not significantly vary across HEP bins ($F_{2,72} = 0.28$, $p = 0.76$). Error bars represent SEMs. ns, not significant.

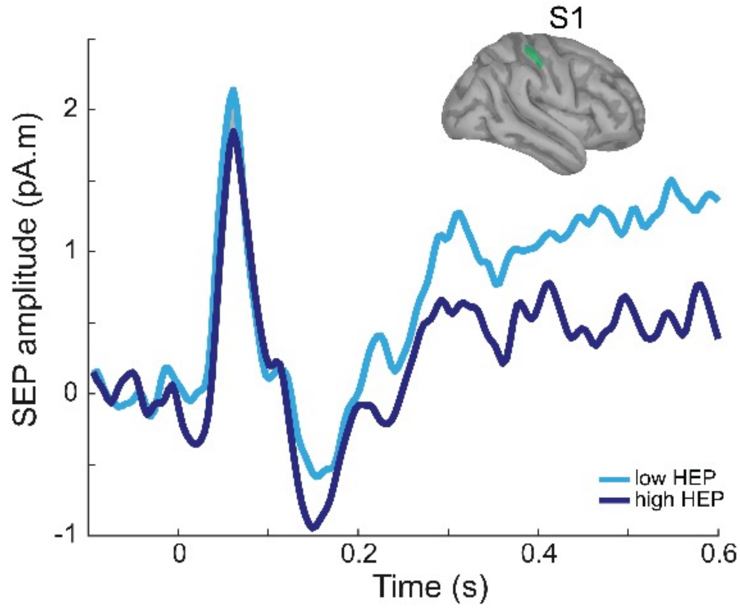

Figure S5. Somatosensory-evoked potentials (SEPs) following low and high HEP amplitudes in source level. A significant difference in P50 amplitude after the low and high HEP amplitudes was observed in the contralateral somatosensory cortex (S1; $t_{36} = 2.15$, $p = 0.03$).

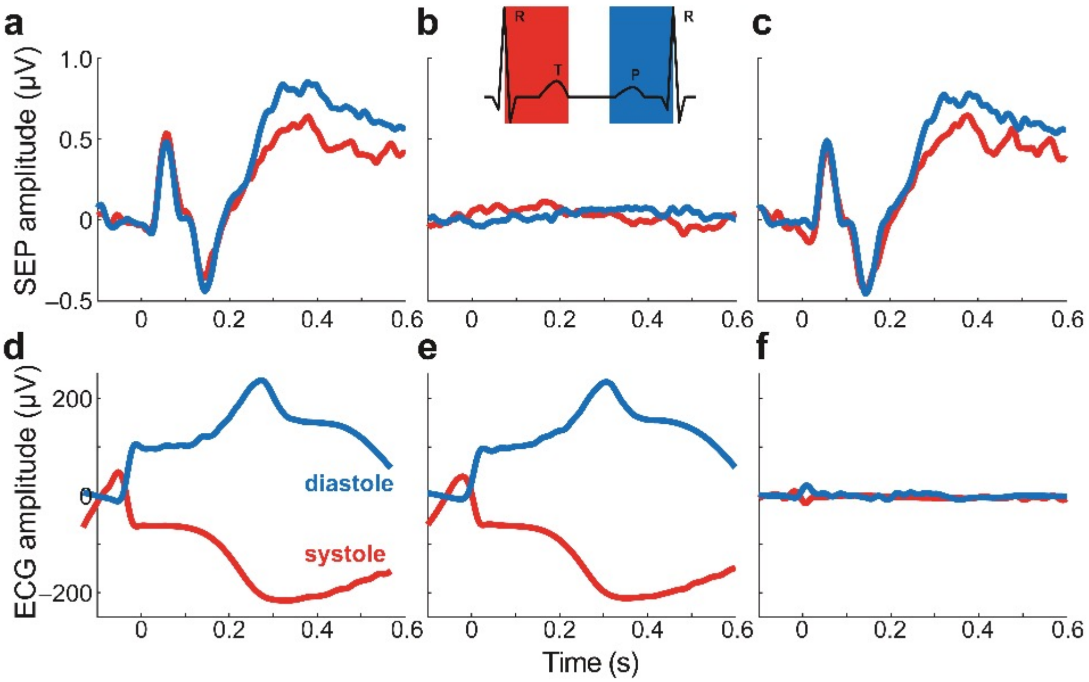

Figure S6. Effect of ECG artifact correction on stimulus-onset locked somatosensory-evoked potential (SEP) and electrocardiogram (ECG) amplitude (0=stimulation onset). (**a**) SEP at C4 before the artifact removal. (**b**) To cancel out the possible effects of ECG artifact, we estimated the cardiac artifact in the evoked responses by first placing random triggers along those cardiac cycles outside the stimulation window of the experiment). Then, we classified the arbitrary triggers as systole or diastole depending on the position of the trigger in the cardiac cycle. After the classification, we segmented data around the triggers and calculated the average cardiac artifact separately for systole and diastole in C4 electrode. (**c**) SEP during systole and diastole after the estimated artifact removal. The average estimation of the cardiac artifact for systole and diastole were subtracted from the SEP separately during systole and diastole. A comparison between uncorrected (a) and corrected SEPs (c) for the ECG artifact indicates that the differences between systole and diastole found between 268−468 ms is observable regardless of the effect of the correction. (**d**) Stimulus onset-locked ECG, grand average across participants before the artifact removal. (**e**) The estimated average cardiac artifacts on ECG amplitude relative to random triggers placed along the cardiac cycles excluding the stimulation window. (**f**) After the subtraction of the estimated cardiac artifact from the stimulus onset-locked ECG activity separately for systole and diastole, the difference in ECG amplitude during diastole versus systole is negligible. This analysis shows that the observed SEP differences between diastole and systole after ECG correction cannot be attributed to differences in cardiac electrical activity.

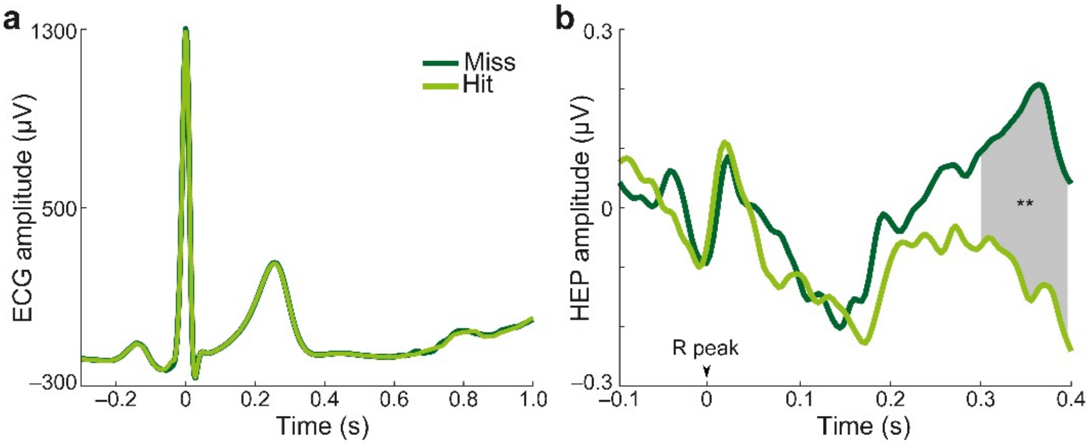

Figure S7. The difference of heartbeat-evoked potential (HEP) amplitude between hits and misses is not due to a cardiac field artifact (**a**) R-peak locked electrocardiogram (ECG) grand average across participants. We did not find any significant difference in ECG data between hits and misses (**b**) The HEP across cluster electrodes before cardiac field artifact removal with independent component analysis. The significant difference of HEP between hits and misses between 296–400 ms (gray area) after the R-peak was conserved. This shows that the artifact correction did not induce changes in the reported HEP-related effects. Furthermore, it suggests that the observed HEP-related effects are not likely to occur due to a volume conduction problem. $^{**}p < 0.005$.

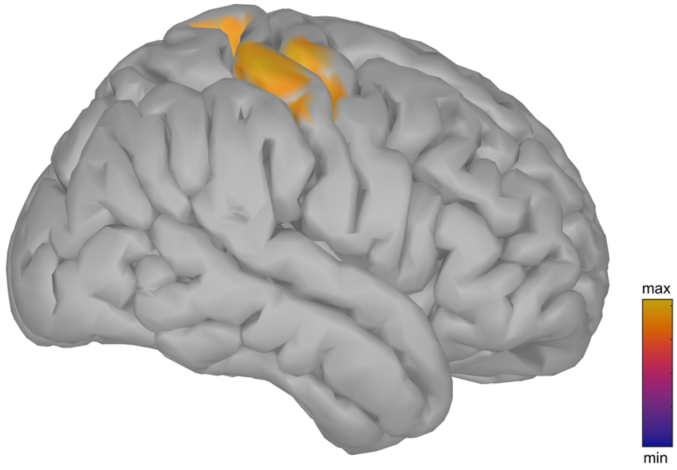

Figure S8. Source localization of sensorimotor ICA components. Sources were reconstructed for ICA components representing sensorimotor rhythms in every subject. Then, the grand average of source activity across subjects was calculated. The figure shows that source activity originates from the contralateral sensorimotor cortex with a maximum in the somatosensory hand area. The visualized activity represents >75% of source strengths.

Table S1. The electrical stimulus intensities (in mA) applied to the index and the middle finger during systole and diastole

	Systole	Diastole	t_{36}	p
Index	2.04 ± 0.64	2.04 ± 0.64	0.35	0.73
Middle	2.26 ± 0.74	2.26 ± 0.75	0.25	0.80
Overall	2.15 ± 0.67	2.15 ± 0.69	0.57	0.57

Table S2 Comparison of mean P300 amplitude between systole and diastole in source level

	t_{36}	p
Anterior Insula (R)	-0.71	0.48
Anterior Cingulate Cortex (R)	1.31	0.20
Anterior Cingulate Cortex (L)	-0.31	0.76
Posterior Cingulate Cortex (R)	2.15	0.04
Posterior Cingulate Cortex (L)	-0.37	0.72
Inferior Parietal Lobule (R)	1.02	0.32
Lateral Prefrontal Cortex (R)	1.06	0.30
Lateral Prefrontal Cortex (L)	1.14	0.26

None of these regions demonstrated significant differences after FDR correction.

Table S3 Comparison of mean P50 amplitudes following low and high HEP amplitudes in source level

	t_{36}	p
**Anterior Insula (R)**	**3.83**	**$5 \cdot 10^{-4}$**
Anterior Cingulate Cortex (R)	-0.17	0.87
Anterior Cingulate Cortex (L)	0.37	0.71
**Posterior Cingulate Cortex (R)**	**-3.39**	**$2 \cdot 10^{-3}$**
**Posterior Cingulate Cortex (L)**	**-4.55**	**$6 \cdot 10^{-5}$**
Inferior Parietal Lobule (R)	1.36	0.18
**Lateral Prefrontal Cortex (R)**	**-4.14**	**$2 \cdot 10^{-4}$**
**Lateral Prefrontal Cortex (L)**	**-3.80**	**$5 \cdot 10^{-4}$**

The highlighted regions demonstrated significant differences after FDR correction.

Table S4 General linear mixed-effects modeling (GLMM) testing the relationship between prestimulus alpha amplitude, cardiac phase, and detection (model no. 1-5)

Model name	Glmer syntax	Likelihood	LRT
1 – $null_1$	detection ~ 1 + (1 | subject)	-14,165	
2 – cardiac	detection ~ cardiac + (cardiac | subject)	-14,156	(1) $\chi 2 = 18.07$***
3 – $alpha_1$	detection ~ alpha + (alpha | subject)	-14,104	(1) $\chi 2 = 121.71$***<br>(2) $\chi 2 = 103.64$***
4 – $additive_1$	detection ~ cardiac + alpha + (cardiac + alpha | subject)	-14,096	(3) $\chi 2 = 17.41$**
5 – $interaction_1$	detection ~ cardiac * alpha + (cardiac * alpha | subject)	-14,095	(4) $\chi 2 = 1.51$

Likelihood shows the log-transformed likelihood of the models. Higher values of likelihood make the model more likely. LRT is the maximum likelihood ratio test comparing two models for the same dataset. More complex models (with more parameters) are compared with respective smaller ones, which gives a *χ2* and *p*-value. **p*< 0.05, ***p*< 0.005, *** *p*< 0.0005.

Table S5 General linear mixed-effects modeling (GLMM) testing the relationship between prestimulus sensorimotor alpha amplitude, heartbeat-evoked potential, and detection (model no. 6-10)

Model name	Glmer syntax	Likelihood	LRT
6 – $null_2$	detection ~ 1 + (1 | subject)	- 10,007	
7 – $alpha_2$	detection ~ alpha + (alpha | subject)	- 9,976	(6) $\chi 2 = 60.27$***
8 – HEP	detection ~ HEP + (HEP | subject)	- 9,964	(6) $\chi 2 = 85.29$***<br>(7) $\chi 2 = 25.02$***
9 – $additive_2$	detection ~ HEP + alpha + (HEP + alpha | subject)	- 9,933	(8) $\chi 2 = 62.73$***
10 – $interaction_2$	detection ~ HEP * alpha + (HEP * alpha | subject)	- 9,932	(9) $\chi 2 = 0.57$

Models are evaluated as in Table 2. *$p< 0.05$, **$p< 0.005$, *** $p< 0.0005$.

References

1. F. Tadel, S. Baillet, J. C. Mosher, D. Pantazis, R. M. Leahy, Brainstorm: A user-friendly application for MEG/EEG analysis. *Comput. Intell. Neurosci.* **2011** (2011).
2. V. Fonov, A. Evans, R. McKinstry, C. Almli, D. Collins, Unbiased nonlinear average age-appropriate brain templates from birth to adulthood. *Neuroimage* **47**, S102 (2009).
3. B. Fischl, FreeSurfer. *Neuroimage* **62**, 774–781 (2012).
4. A. Gramfort, T. Papadopoulo, E. Olivi, M. Clerc, OpenMEEG: opensource software for quasistatic bioelectromagnetics. *Biomed. Eng. Online* **9**, 45 (2010).
5. R. D. Pascual-Marqui, Discrete, 3D distributed, linear imaging methods of electric neuronal activity. Part 1: exact, zero error localization (2007) (January 17, 2020).
6. C. Destrieux, B. Fischl, A. Dale, E. Halgren, Automatic parcellation of human cortical gyri and sulci using standard anatomical nomenclature. *Neuroimage* **53**, 1–15 (2010).
7. D. Bates, M. Mächler, B. Bolker, S. Walker, Fitting Linear Mixed-Effects Models Using lme4. *J. Stat. Software; Vol 1, Issue 1* (2015) https:/doi.org/10.18637/jss.v067.i01.
